# Cross-Cultural Comparison of the Contexts Associated with Emotional Outbursts

**DOI:** 10.1007/s10803-022-05708-7

**Published:** 2022-08-19

**Authors:** Justin Cheuk Yin Chung, Rosane Lowenthal, Carmel Mevorach, Cristiane Silvestre Paula, Maria Cristina Triguero Veloz Teixeira, Kate Anne Woodcock

**Affiliations:** 1 School of Psychology, University of Birmingham, Birmingham, B15 2SA UK; 2 Santa Casa de São Paulo School of Medical Sciences, São Paulo, Brazil; 3 Developmental Disorders Program and Mackenzie Center for Research in Childhood and Adolescence at Mackenzie Presbyterian University, São Paulo, Brazil; 4 Centre for Applied Psychology, School of Psychology & Institute for Mental Health, University of Birmingham, Birmingham, UK

**Keywords:** Emotional outburst, Temper outburst, Neurodevelopmental disorder, Cultural difference

## Abstract

The causal relationship between emotional outbursts and emotion dysregulation is proposed to be heterogeneous, but cultural influences have not been considered despite established cultural differences in emotional processes (e.g., increased motivation to suppress emotions in interdependent cultures). Responses to the Brazilian Portuguese version of the Emotional Outburst Questionnaire were collected from 327 caregivers of young people (6–25 years) with autism spectrum disorder, Down’s syndrome, or intellectual disability. Responses were compared to a previous sample of 268 responses from the English version of the questionnaire. The latent factor structure of the contextual items was measurement invariant across both versions. The Brazilian responses were classified into three distinct clusters (Sensory Sensitivity; Perceived Safety; Perceived Unsafety) which considerably overlapped with the English clusters.

**Supplementary Information:**

The online version contains supplementary material available at 10.1007/s10803-022-05708-7.

## Background

Emotional outbursts (also known as “temper outbursts”) are one of the most common forms of challenging behaviours in people with autism spectrum disorder (ASD) and other neurodevelopmental disorders, with the potential to cause a wide range of negative impacts on the quality of life of people experiencing outbursts and their families (Acker et al., 2018; Lowe & Felce, 1995; Lowe et al., 2007; Montaque et al., 2018; Myrbakk & Tetzchner, 2008; Ryan, 2010). Previous studies have revealed large variability in terms of the contexts associated with outbursts, which comprise of discrete events with the potential to directly trigger outbursts (antecedents) and background factors that may increase the likelihood for individuals to experience outbursts (setting events; Beauchamp-Châtel et al., 2019; Cressey et al., 2019; Rice et al., 2018; Tunnicliffe et al., 2014).

Emotional outbursts are commonly attributed to a person’s underlying difficulties with emotion regulation or expression (i.e., emotion dysregulation). For example, the experience of emotional outbursts constitutes one item within the Emotion Dysregulation Inventory, which has been specifically developed for measuring dysregulation in individuals with ASD (Mazefsky et al., 2018). However, this one-to-one correspondence appears to be an oversimplification of the link between emotion dysregulation and outbursts, as the interactions between processes such as cognitive, social, and environmental influences contribute to a high degree of heterogeneity in both phenomena, a feature which may be similarly reflected in their causal relationship (Acker et al., 2018; Aldao et al., 2015; Astle et al., 2022; Colombo et al., 2020; Tunnicliffe & Oliver, 2011; Tunnicliffe et al., 2014; White et al., 2014; Woodcock et al., 2011). Therefore, it is possible that the manifestation of emotional outbursts may vary within and across individuals through multiple distinct pathways, owing to the different ways in which people may be emotionally dysregulated. On the basis that the contexts associated with emotion dysregulation and subsequent emotional outbursts are a common source of variability for both phenomena, we propose that people may experience emotional outbursts in specific subsets of contexts due to underlying differences in emotion regulation or expression that may be unique to those contexts.

Whilst the current literature is lacking, a step towards developing an aetiological account of emotional outbursts is to identify any different patterns of contexts in which individuals experience outbursts, which can subsequently shed light on the underlying differences in emotion regulation or expression associated with those contexts. For example, a previous study involving the caregivers of people with Lowe syndrome has reported a distinct absence of emotional outbursts outside of the home, despite frequent endorsement of antecedents that could commonly occur both in and out of the home environment (e.g., change in own routine), which has similarly been observed in some anecdotal reports by caregivers of people with other neurodevelopmental conditions (Cressey et al., 2019). Under our current proposal, this type of context-dependence of emotional outbursts for some people may be the consequence of differences in emotion regulation or expression which are similarly specific to certain contexts, such that these differences may inhibit the manifestation of outbursts outside of the home and/or facilitate the manifestation of outbursts within the home. Following this line of inquiry may be especially informative for the development of effective interventions for emotional outbursts, as the emotional differences that lead to outbursts within specific contexts can be more directly targeted.

### The Aetiological Framework of Emotional Outbursts

Towards the goal of identifying the different patterns of contexts associated with outbursts that may be aetiologically relevant, an exploratory study developed the Emotional Outburst Questionnaire and collected responses from caregivers of children and young people (Chung et al., 2022). The development of this new measure was argued as necessary for advancing our understanding of emotional outburst aetiology, as other existing measures collect limited information regarding outbursts (e.g., frequency of outbursts in the Developmental Behaviour Checklist; Einfeld & Tonge, 1995), or were developed for typically developing individuals (e.g., the Multidimensional Assessment of Preschool Disruptive Behavior; Wakschlag et al., 2012, 2014). Using the Emotional Outburst Questionnaire, it was found that the majority of antecedents and setting events of emotional outbursts could be organised into six latent factors (Chung et al., 2022). These factors were labelled as (1) Sensory, which included sensory stimuli (e.g., sudden or loud noises); (2) Cognitive Demand, which contained antecedents that may be cognitively demanding for an individual (e.g., change in expectation); (3) Threat to Self, which consisted of antecedents that may threaten the self-esteem of an individual (e.g., being criticised); (4) Cross-settings, which contained a range of setting events (e.g., public setting, or unfamiliar person); (5) Safety, which included setting events perceived as safe (e.g., private setting); (6) States, which consisted of setting events related to the physiological state of the individual (e.g., tired).

Based on these contextual factors, responses were transdiagnostically classified into three unique clusters, such that people who experienced outbursts in a similar pattern of contexts were grouped together. These three clusters were proposed to each represent an aetiological pathway to emotional outbursts: (1) children and young people with differences in sensory processing may be more susceptible to experience outbursts across antecedents and setting events due to the additional demand of background stimuli (the Sensory Sensitivity pathway); (2) children and young people who mask their distress in settings that lack perceived safety may experience more outbursts upon returning to safe setting events due to the build-up of distress and the cost of masking (the Perceived Safety pathway); (3) children and young people with differences in safety perception may consider more environments to lack perceived safety, thereby increasing the likelihood for outbursts to be experienced in these unsafe setting events (the Perceived Unsafety Pathway; Chung et al., 2022). As this framework of contextual pathways was derived from exploratory and inductive analysis, the validity and replicability of this proposal remain untested. Therefore, the potential generalisability of the previous interpretations requires evaluation through confirmatory analysis with additional data.

### The Impact of Culture on Emotion Regulation and Emotional Outbursts

The need for additional sampling presents a further opportunity to explore the potential influence of culture on the aetiology of emotional outbursts, as the pathways proposed in the previous study were largely constrained to the context of English-speaking caregivers from the United Kingdom. Cross-cultural studies have commonly investigated the differences in emotional processes between societies that promote the independence of the self from others (e.g., the UK) versus societies that prioritise the interdependence of the self with others (e.g., Brazil; Markus & Kitayama, 1991, 2010). These studies have revealed differing cultural norms in the regulation and expression of emotions (for reviews, see Ford & Mauss, 2015; Friedlmeier et al., 2014), which may lead to fundamental differences in the manifestation and experience of emotional outbursts in individuals across cultures. For example, it has been demonstrated that people from relatively interdependent cultures were more likely to suppress emotions compared to people from relatively independent cultures, a tendency which was motivated by the desire to maintain harmony across interpersonal relationships (Matsumoto et al., 2008; Ramzan & Amjad, 2017). Indeed, this degree of interdependence is apparent in countries such as Brazil, as Brazilian families tend to avoid conflict and place particular emphasis on pleasing family, friends, and the wider community (Dessen & Torres, 2019). Thus, it is possible that individuals from relatively interdependent cultures such as Brazil may be more likely to suppress their distress in response to antecedents, especially within contexts that involve other people (e.g., in public), thereby increasing the likelihood of emotional outbursts manifesting via the Perceived Safety pathway. Additionally, families of individuals with ASD in Brazil have reported a lack of support to socially integrate individuals with ASD into the wider community; and inadequate measures to minimise the exposure to intimidating environments for individuals with ASD (Gomes et al., 2015; Weissheimer et al., 2021). Consequently, these two factors may contribute to the lack of perceived safety in contexts involving the wider community (Dammeyer & Chapman, 2018), further contributing to the experience of distress in these environments.

In addition to cultural differences in how individuals respond to antecedents, there may be further cultural differences in the caregivers’ response that can further modulate the likelihood of emotional outbursts in different contexts. For example, cultural differences in the beliefs around the acceptability of expressing negative emotions appeared to influence how caregivers from Brazil and other countries preferred to respond in situations that elicited such emotions in their children (Corapci et al., 2018; Mograbi et al., 2018; Rimes & Chalder, 2010). Cultural differences in the experience of stigma for caregivers of individuals with ASD and other neurodevelopmental disorders may further influence caregiver response to negative emotion expression (Kinnear et al., 2016). For example, a pioneering study covering a large sample of caregivers of people with ASD across Latin America reported that Brazilian caregivers experienced significantly more stigma compared to caregivers from other Latin American countries, which could be related to the greater frustration Brazilian caregivers experienced when seeking support and the lower verbal ability of people with ASD in the Brazilian sample (Paula et al., 2020). Therefore, caregiver response to emotional outbursts may be differentiated by cultural variations in beliefs around negative emotion expression and the experience of stigma, which may include context-specific differences in the caregiver response (e.g., at home versus in public) that can conceivably promote or suppress the experience of emotional outbursts by individuals in different contexts.

As such, the consideration of culture is critical for the proposed framework, as culture itself could be deemed as an overarching level of context that may directly influence how setting events and antecedents may be organised into latent factors, and how these factors may in turn relate to the aetiology of emotional outbursts (Bronfenbrenner & Morris, 2006). There may be fundamental differences in the pathways that emerge from the responses of a culturally distinct sample, in terms of the distinguishing contexts and mechanisms associated with the identified pathways, and the demographic composition of each group. If a pathway were indeed found to be invariant across cultures, this would demonstrate the importance of recognising the significance of the associated contexts when considering the mechanisms underlying current and potential interventions for emotional outbursts. From a measurement perspective, it would be critical to assess the cross-cultural invariance of the measured constructs related to emotional outbursts, as this would inform the potential generalisability of the derived framework. Furthermore, recent work suggested that measures of emotion regulation strategies were largely invariant across samples of adults from the US and India (Van Doren et al., 2021). If a similar degree of invariance were found across cultures for measures of emotional outbursts, this could facilitate future endeavours in expanding the present framework by integrating patterns of emotional regulation strategies in this cross-cultural aetiological account of emotional outbursts.

The present study aimed to investigate whether the contextual clusters of emotional outbursts that emerge from a culturally distinct sample of caregivers in Brazil would be comparable to the previously identified clusters. To facilitate this cross-cultural comparison, the Emotional Outburst Questionnaire was translated and culturally adapted into a Brazilian Portuguese version, and the factor structure of the contextual items in the Brazilian Portuguese version of the questionnaire was validated and compared against the factors derived from the English version.

## Methods

### Participants

Caregivers of young people (aged 6–25 years) who experienced emotional outbursts at least once a month were recruited via local autism units for families of children and young people with neurodevelopmental conditions in Brazil. In addition to these units, the questionnaire was also available on social networks of the authors MCTVT and RL for recruitment of eligible caregivers. The age and outburst frequency inclusion criteria matched those of the previous English study (Chung et al., 2022). Out of a total of 359 completed responses, one participant was excluded as the young person was outside the age inclusion criterion. To ensure that the final sample met the criterion for outburst frequency, 27 participants were excluded based on their response to the question regarding general outburst frequency (item 57) on the Emotional Outburst Questionnaire: 20 indicated Less than once a month; six selected Never; one response was missing. Four participants were excluded as their responses were partially missing for items regarding the antecedents and setting events of emotional outbursts (items 58–112).

Out of the final sample of 327 responses, 250 young people were male (76.5%) and 77 were female (23.5%). The mean age of the young people in the sample was 10.7 years (*SD* = 3.7; range = 6.0–25.7; Supplementary Fig. 1). Caregivers provided information regarding diagnoses of ASD, Down’s syndrome, and/or intellectual disability in a multiple-choice question format. Information regarding other diagnoses was not collected. Across the sample, 252 caregivers selected only ASD (77.1%); 37 selected only Down’s syndrome (11.3%); 11 selected only intellectual disability (3.4%); eight selected ASD and Down’s syndrome (2.4%); 12 selected ASD and intellectual disability (3.7%); six selected Down’s syndrome and intellectual disability (1.8%); no diagnosis was selected by one caregiver (0.3%). Therefore in total, 272 young people had a diagnosis of ASD (83.2%); 51 individuals were diagnosed with Down’s syndrome (15.6%); 29 young people had an intellectual disability (8.9%).

Caregivers of 107 young people indicated that medication was taken for emotional outbursts (32.7%). Fifty-four families have accessed support for outbursts in terms of a programme, intervention, or training (16.5%), 48 of whom found the support to be effective (88.9%). Of the families who have accessed support, 10 received this support immediately or soon after difficulties with outbursts began (18.5%), whilst the 44 remaining families received support a while after (81.5%). With regards to schooling or employment status, 262 young people were in mainstream schools (80.1%); 51 attended special schools (15.6%); one was in further education (0.3%); one was in higher education (0.3%); four were in employment preparation (1.2%); seven were unemployed (2.1%; one missing). Overall, 194 young people received a statement of special educational needs or have an educational plan at school (59.3%). Twenty-five caregivers reported that the young people they were caring for have experienced early traumatic or adverse events (7.6%; four selected *Prefer not to say*; 11 missing), which were described to encompass: natural disasters; death or serious injury of someone close to the person; poverty; witnessing abuse or violence; emotional, physical, or sexual abuse; neglect. Supplementary Table 1 compares the demographic information between caregivers from the English (Chung et al., 2022) and Brazilian samples. Differences were present across demographic variables. Compared to the English sample, the Brazilian sample had: a lower mean age; a higher proportion of boys; a higher proportion of diagnoses of ASD and Down’s syndrome; a lower proportion of diagnosis of intellectual disability; a higher proportion of pharmacological intervention use for outbursts; a lower proportion of non-pharmacological intervention use for outbursts; differences in schooling and employment status; and a lower proportion of exposure to trauma.

### Measures

The Emotional Outburst Questionnaire is an informant-report measure, which contains 133 items pertaining to the characteristics of outbursts, including the antecedents and setting events associated with outbursts, in addition to frequency, duration, intensity, and behaviours observed in the most and least severe outbursts (Chung et al., 2022). The 55 items related to the antecedents and setting events of outbursts were rated on a three-point scale: *Not applicable/never/rarely (0–3 times out of 10)*, *Sometimes (4–6 times out of 10)*, *Often/always (7–10 times out of 10)*. In the English study, most contextual items loaded onto six latent factors, with internal consistency as indicated by Cronbach’s α ranging from 0.68 to 0.84 (Chung et al., 2022).

The translation and cross-cultural adaptation of the Brazilian Portuguese Emotional Outburst Questionnaire was carried out in a previous study (Balbueno, 2021), following the stages recommended by the International Test Commission (2017). The process included: (1) independent translations by two native speakers of Brazilian Portuguese with proficiency in English; (2) synthesis of the two translations and analysis of the synthesis by three experts (two native speakers of Brazilian Portuguese and one native speaker of English) that compared the synthesis with the original versions of the translations using recommendations of the International Test Commission (2017); (3) analysis by the participants of the target audience based on the understanding of the items, clarity and accuracy; (4) back-translation by a native English speaker and final modifications to rectify discrepancies between the original and back-translated versions of the measure (Balbueno, 2021).

### Procedure

Caregivers provided informed consent prior to participating in this study. The majority of participants completed the Brazilian Portuguese version of the Emotional Outburst Questionnaire online on the Research Electronic Data Capture (REDCap) platform (Harris et al., 2009, 2019). Twenty responses were collected in person from caregivers attending a local mental health care centre. Questionnaire and demographic data for the 268 responses to the English version of the questionnaire were accessed via the publicly available repository: https://osf.io/2j47e/.

### Statistical Analyses

#### Confirmatory Factor Analysis

The validity of the factor structure of the contextual items derived from the English data was evaluated with confirmatory factor analysis in the present sample. The factor structure of the base model consisted of items with factor loadings ≥ 0.40 in the previous exploratory factor analysis (Supplementary Fig. 2; Chung et al., 2022). A series of confirmatory models was estimated using the diagonally weighted least squares method with delta parametrisation in R 4.0.2 (R Core Team, 2021) with the lavaan package (Rosseel, 2012). Model fit was determined by evaluating multiple measures: chi-square statistic, comparative fit index (CFI), root-mean-square error of approximation (RMSEA) and its 90% confidence intervals, and standardised root-mean-square residual (SRMR). Good model fit is indicated by non-significant chi-square statistic, CFI > 0.95, RMSEA < 0.06, and SRMR < 0.08 (Hu & Bentler, 1999). However, the chi-square statistic is sensitive to sample size, such that chi-square tests with larger samples are more likely to be significant (Schermelleh-Engel et al., 2003). Acceptable model fit is indicated by CFI > 0.90, RMSEA < 0.08, and SRMR < 0.10 (Kline, 2016; van de Schoot et al., 2012). After each model was fitted, modification indices were examined to identify changes to model specification that may improve model fit (Kline, 2016). Each model subsequent to the base model included the addition of a modification which was theoretically relevant and expected to have a high impact on model fit. Internal consistency for each factor in the final model is reported in terms of Cronbach’s α. Consistent with the analysis of the previous English study, refined and non-refined factor scores were calculated according to the final confirmatory factor model for each participant for subsequent cluster analyses (Chung et al., 2022). Standardised regression-based factor scores, which accounted for factor loadings and intercorrelations, represented the refined factor scores. In contrast, non-refined factor scores involved taking an unweighted average of the responses to items loading onto each factor.

#### Measurement Invariance Analysis

Following identification of the final model, the responses collected with the Brazilian Portuguese version of the Emotional Outburst Questionnaire were compared to those collected with the English version in a multiple-group confirmatory factor analysis to evaluate measurement invariance of the items involved in this factor structure. Establishing measurement invariance across the two groups would allow for between-group comparisons of contextual items and factors. As per the considerations for measurement invariance in ordered categorical items by Wu and Estabrook (2016), the degree of measurement invariance was evaluated through a series of models with increasing equality constraints across the two groups: (1) configural invariance was tested by fitting both groups to an identical factor structure; (2) threshold invariance included constraining the thresholds for response categories of each item to be equal across the two groups, in addition to specifying an identical factor structure; (3) loading invariance consisted of constraining factor loadings to be equivalent across the two groups, in addition to the constraints of the threshold invariance model. Measurement invariance models were fitted using the diagonally weighted least squares estimator with delta parametrisation, in accordance with the procedures outlined by Svetina et al. (2020), using R packages lavaan (Rosseel, 2012) and semTools (Jorgensen et al., 2021). The changes in model fit indices across models were evaluated to assess measurement invariance. Threshold invariance and loading invariance are indicated by changes in SRMR (ΔSRMR) that are less than 0.010 and 0.030, respectively, in addition to ΔCFI <  − 0.010 and ΔRMSEA < 0.015 (Chen, 2007).

#### Cluster Analysis of Brazilian Refined Factor Scores

The refined factor scores for the responses from caregivers in Brazil were used to explore patterns of contexts in which outbursts occurred through cluster analyses. The procedure of the previous English study was followed to identify clusters with a data-driven approach, such that the process was independent of the previous outcomes (Chung et al., 2022). The refined factor scores of caregivers in Brazil first underwent hierarchical agglomerative clustering (Ward, 1963), in which three clusters were identified as an appropriate cluster structure with interpretable clusters. Subsequently, the refined factor scores were analysed with *k*-means clustering with *k* = 3, which allowed for the mean factor scores of each cluster (centroid) to be specified and compared. Classification agreement between hierarchical and *k*-means clustering was achieved for 247 responses [75.5%; Cohen’s unweighted κ = 0.64, 95% CI (0.57, 0.71)]. The mean scores of the contextual factors were compared between clusters in a multivariate analysis of variance, followed by separate univariate Welch’s ANOVA and post-hoc pairwise Games-Howell tests. Demographic variables with sufficient data were compared between clusters with χ^2^ tests of association and ANOVA.

#### Cross-Cultural Comparison of Brazilian and English Clusters

To identify cross-cultural similarities and differences in the clusters between the two samples, the mean factor scores of each cluster derived from the Brazilian sample were compared to the previously reported mean factor scores of the corresponding cluster from the English sample using Welch’s t-tests.

#### Cluster Analysis of Brazilian Non-Refined Factor Scores

Non-refined factor scores of Brazilian responses were subject to *k*-means cluster analysis to examine whether these simplified factor scores could produce similar results to those derived from refined factor scores. Establishing invariance in cluster membership across refined and non-refined factor scores would suggest that non-refined factor scores could be used for the clustering of future responses. The use of non-refined factor scores would be preferable over refined scores, as non-refined scores could be calculated for subsequent samples without the requirement of additional factor analysis. When the cluster structures derived from *k*-means clustering of refined factor scores and *k*-means clustering of non-refined factor scores were compared, cluster membership was retained for 272 responses (83.2%; Cohen’s unweighted κ = 0.74, 95% CI [0.68, 0.80]). As with the analyses conducted for refined factor scores, the mean non-refined factor scores were compared via MANOVA, Welch’s ANOVA, and Games-Howell tests.

#### Cross-Cultural Comparison of Methods of Cluster Classification

A final set of analyses was performed to evaluate whether the mean scores of non-refined clusters reported in the English study could be utilised as a point of reference to reliably classify the Brazilian responses into these established clusters. If this were demonstrated to be the case, it would support the notion that these cluster centroids may be sample invariant, such that new responses could be classified into these clusters without the need to perform further cluster analysis, thereby allowing for the use of the measure as a screening tool for small numbers of responses. To achieve this goal, the cluster structure based on the non-refined factor scores of the Brazilian responses was compared to classification of the Brazilian responses using cluster centroids of non-refined factor scores from the English study. In the latter procedure, each response from the Brazilian sample was assigned to one of the clusters from the English study with the nearest centroid in terms of Euclidean distance. Classification agreement between these two methods represented a measure of the ability for the cluster centroids established in the English study to classify new responses.

## Results

### Validation of English Contextual Factors in the Brazilian Portuguese Emotional Outburst Questionnaire

The six-factor solution from the previous analyses (Supplementary Fig. 2) was fitted onto the Brazilian responses, which resulted in inadequate model fit based on the chi-square statistic, RMSEA, and SRMR, but acceptable fit based on the CFI [χ^2^ (512) = 2444.080, p < 0.001; CFI = 0.930; RMSEA = 0.108, 90% CI (0.103, 0.112); SRMR = 0.119]. The modification indices of this base model identified that the addition of the item ‘Familiar person’ loading onto the Safety factor would improve model fit by the greatest amount. With the addition of this loading, the model demonstrated similar levels of fit as the base model [χ^2^ (5110 = 2028.165, p < 0.001; CFI = 0.945; RMSEA = 0.095, 90% CI (0.091, 0.100); SRMR = 0.110]. An examination of the modification indices for this model indicated that ‘Familiar setting’ additionally loading onto the Safety factor would further improve model fit. Indeed, addition of this loading improved model fit as indicated by the CFI, RMSEA, and SRMR to levels ranging from acceptable to good [χ^2^ (510) = 1523.689, p < 0.001; CFI = 0.963; RMSEA = 0.078, 90% CI (0.074, 0.083); SRMR = 0.100]. Therefore, the final model differed from the base model in terms of the additional loading of two items (‘Familiar person’ and ‘Familiar setting’) onto the Safety factor (Supplementary Fig. 3). The internal consistency of each factor, as measured by Cronbach’s α was found to be similar to the previous analyses (Sensory = 0.76; Cognitive Demand = 0.81; Threat to Self = 0.82; Cross-settings = 0.80; Safety = 0.85; States = 0.84).

### Measurement Invariance Across the English and Brazilian Portuguese Emotional Outburst Questionnaire

When the responses from the English (*N* = 268) and Brazilian Portuguese (*N* = 327) versions of the questionnaire were combined and fitted onto the final model, the CFI, RMSEA, and SRMR indicated acceptable to good fit [χ^2^ (1020) = 2636.512, p < 0.001; CFI = 0.959; RMSEA = 0.073, (0.070, 0.077); SRMR = 0.098], demonstrating configural invariance across the two versions of the questionnaire. As each contextual item comprised of three response options, the threshold invariance model demonstrated no change in fit indices, because the configural invariance and threshold invariance models were statistically equivalent under this condition (Wu & Estabrook, 2016). The fit of the loading invariance model was found to be similar to the previous model [χ^2^ (1050) = 2786.788, p < 0.001; CFI = 0.956; RMSEA = 0.075, (0.071, 0.078); SRMR = 0.099], such that the changes in the fit indices across the models suggested that loading invariance was demonstrated across the two versions of the questionnaire (ΔCFI = 0.003; ΔRMSEA = 0.002; ΔSRMR = 0.001). Taken together, the measurement invariance analysis indicated that the information regarding the contexts of outbursts captured by the two versions of the questionnaire could be represented by an identical factor structure with the same set of factor loadings.

### Contextual Clusters of the Brazilian Responses

#### Refined Factor Scores

**Fig. 1:**
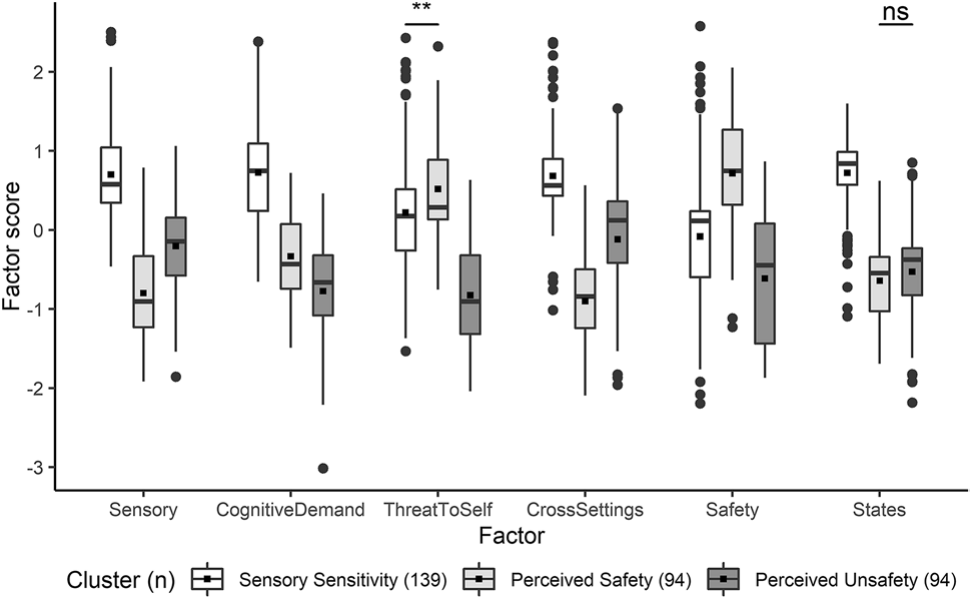
Pairwise comparisons of the three clusters derived from Brazilian refined factor scores. Boxplots show mean (black squares) median (horizontal bar), interquartile range (box), range (whiskers) and outliers (circles). All outliers were included in analyses. Unless otherwise specified, all pairwise comparisons within each factor were significant at p < 0.001, adjusted with Tukey’s method, *Ns* not significant; **p < 0.01

The refined factor scores of the responses based on the Brazilian Portuguese questionnaire were clustered into three groups. The clusters closely resembled those derived from the previous English study (see below), and were therefore accordingly labelled as the Sensory Sensitivity, Perceived Safety, and Perceived Unsafety clusters. There was a significant difference in the mean refined factor scores between the clusters [Pillai Trace = 0.597, *F*(6, 320) = 79.019, *p* < 0.001, ω^2^ = 0.589, 95% CI (0.508, 0.648)], and the univariate tests revealed that there were significant differences between the clusters in the mean scores across all six factors (Table 1). All but one of the pairwise comparisons of the mean factor scores were significantly different (Fig. 1; Supplementary Table 2). In the Sensory Sensitivity cluster, the mean scores were relatively high across the factors. The mean scores of the Threat to Self and Safety factors were highest in the Perceived Safety cluster. The mean scores of the Perceived Unsafety cluster were relatively low across factors, but the Sensory and Cross-settings scores for this cluster appeared to be moderately high.

**Table 1:** Cluster centroids of the three-cluster solution derived from Brazilian refined factor scores

Factor	Cluster mean (*SD*)			Welch’s *F*	ω^2^	95% CI	Post-hoc summary^a^
	SS (*n* = 139)	PS (*n* = 94)	PU (*n* = 94)				
Sensory	0.70 (0.58)	− 0.80 (0.61)	− 0.20 (0.58)	*F*(2, 199) = 185.4***	0.530	[0.389, 0.645]	1 > 3 > 2
Cognitive demand	0.73 (0.61)	− 0.33 (0.51)	− 0.77 (0.65)	*F*(2,202) = 184.6***	0.529	[0.390, 0.643]	1 > 2 > 3
Threat to Self	0.22 (0.80)	0.52 (0.57)	− 0.83 (0.69)	*F*(2, 209) = 111.4***	0.403	[0.259, 0.532]	2 > 1 > 3
Cross-settings	0.68 (0.55)	− 0.90 (0.55)	− 0.12 (0.75)	*F*(2, 191) = 231.5***	0.585	[0.449, 0.692]	1 > 3 > 2
Safety	− 0.08 (0.79)	0.72 (0.69)	− 0.61 (0.76)	*F*(2, 204) = 81.6***	0.330	[0.187, 0.466]	2 > 1 > 3
States	0.72 (0.50)	− 0.64 (0.48)	− 0.53 (0.65)	*F*(2, 193) = 263.0***	0.616	[0.486, 0.716]	1 > 2, 3

*SS* sensory sensitivity; *PS* perceived safety; *PU* perceived unsafety, p and confidence intervals adjusted with Bonferroni correction, ***p < 0.001

^a^Pairwise Games-Howell tests adjusted with Tukey’s method

Demographic variables for which sufficient data was available for statistical comparison are presented for each cluster in Table 2. There was no significant difference between the clusters in terms of age, gender, or diagnosis of the young person. Significant differences between the clusters were found in terms of whether the young person received medication for emotional outbursts and whether the family have had access to non-pharmacological support for outbursts. Post-hoc pairwise comparisons revealed that a lower proportion of individuals in the Perceived Safety cluster received medication for outbursts compared to the Sensory Sensitivity cluster [χ^2^ (1) = 29.77, p < 0.001, Cramer’s V = 0.37, 95% CI (0.23, 0.48), adjusted with Bonferroni correction] and the Perceived Unsafety cluster [χ^2^ (1) = 15.70, p < 0.001, Cramer’s V = 0.30, 95% CI (0.14, 0.45), adjusted with Bonferroni correction], whereas a higher proportion of individuals in the Perceived Safety cluster have had access to other forms of support for outbursts compared to the Sensory Sensitivity cluster [χ^2^ (1) = 8.83, p = 0.009, Cramer’s V = 0.21, 95% CI (0.04, 0.360), adjusted with Bonferroni correction].

**Table 2:** Demographics of each of the three clusters derived from Brazilian refined factor scores

Variable^a^	Cluster			Statistic	Effect size^b^	95% CI
	SS	PS	PU			
*N*	139	94	94			
Age						
Mean	10.9	10.5	10.4	*F*(2, 324) = 0.64	0.00	[0.00, 1.00]
*SD*	3.5	4.02	3.68			
Gender (%)				χ^2^ (2) = 1.27	0.06	[0.02, 0.19]
Male	77.7	78.7	72.3			
Female	22.3	21.3	27.7			
Diagnosis (%)						
ASD	87.8	80.9	78.7	χ^2^ (2) = 3.79	0.11	[0.03, 0.23]
DS	12.9	13.8	21.3	χ^2^ (2) = 3.27	0.10	[0.03, 0.23]
I D	5.8	13.8	8.5	χ^2^ (2) = 4.54	0.12	[0.03, 0.23]
Medication (%)						
Yes	45.3	10.6	36.2	χ^2^ (2) = 31.36***	0.31	[0.23, 0.40]
Access to support (%)						
Yes	11.5	27.7	12.8	χ^2^ (2) = 11.95**	0.19	[0.08, 0.31]

*SS* sensory sensitivity; *PS* perceived safety; *PU* perceived unsafety; *ASD* autism spectrum disorder; *DS* Down’s syndrome; *ID* intellectual disability, **p < 0.01; ***p < 0.001

^a^Experiernce of early traumatic or adverse events could not be reliably compared across clusters due to low endorsement rate

^b^ω2 for ANOVA and Cramer’s V for χ2 tests

**Fig. 2:**
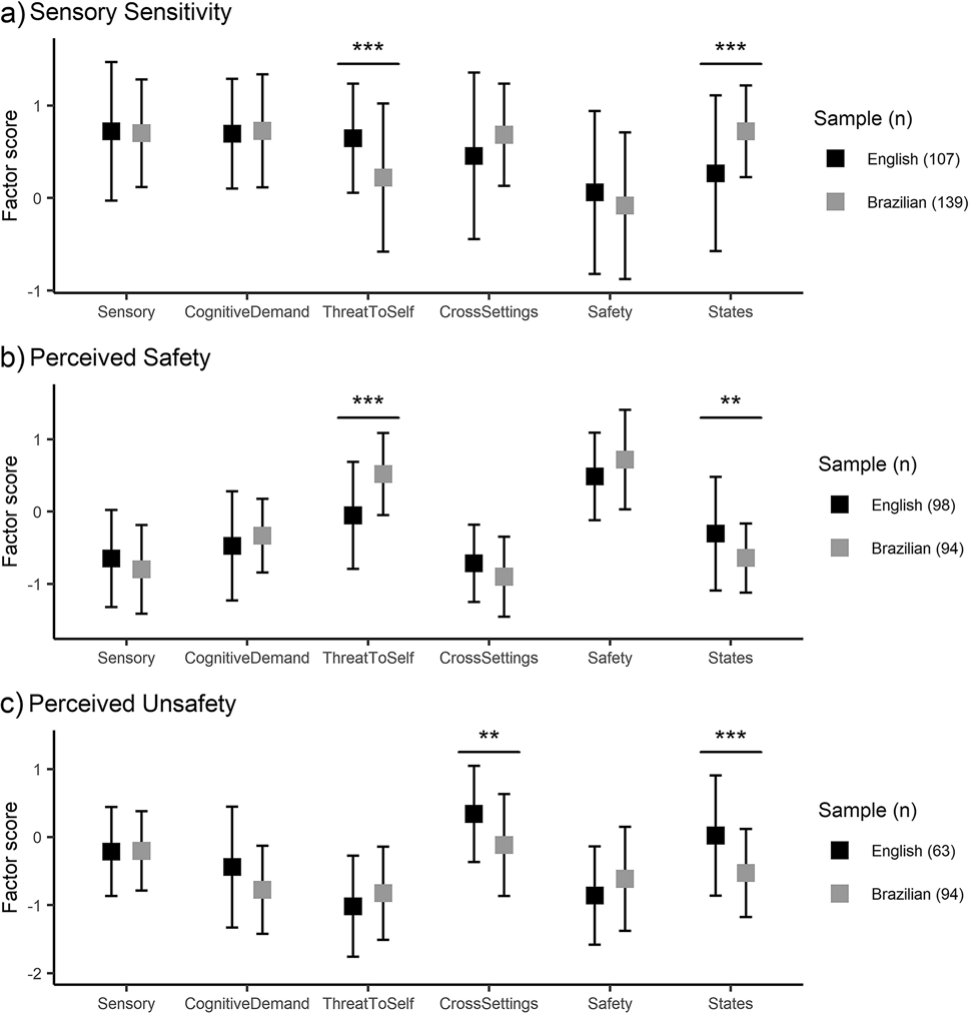
Centroids of **a** the Sensory Sensitivity, **b** Perceived Safety, and **c** Perceived Unsafety clusters derived from refined factor scores from the samples using the English (black squares) and Brazilian Portuguese (grey squares) versions of the Emotional Outburst Questionnaire, Error bars represent standard deviations. p adjusted with Bonferroni correction across all comparisons, **p < 0.01; ***p < 0.001

The mean factor scores of each cluster from the Brazilian responses were largely comparable to the mean factor scores of the corresponding cluster from the English responses (Fig. 2). For the Sensory Sensitivity cluster, significant differences between the samples were found in two factors: in the Brazilian sample, the mean Threat to Self score was lower [*t*(244) = -4.82, *p* < 0.001, Cohen’s *d* = -0.607, 95% CI (− 1.15, − 0.20), adjusted with Bonferroni correction] and the mean States score was higher [*t*(161) = 4.97, *p* < 0.001, Cohen’s *d* = 0.659, 95% CI (0.26, 1.08), adjusted with Bonferroni correction]. The mean Threat to Self and States scores were also significantly different between the samples for the Perceived Safety cluster: in the sample using the Brazilian Portuguese version of the questionnaire, the mean Threat to Self score was higher [*t*(182) = 6.01, *p* < 0.001, Cohen’s *d* = 0.866, 95% CI (0.48, 1.27), adjusted with Bonferroni correction] and the States score was lower [*t*(161) = -3.61, *p* = 0.007, Cohen’s *d* = − 0.518, 95% CI (− 0.95, − 0.06), adjusted with Bonferroni correction]. The mean Cross-settings [*t*(138) = -3.88, *p* = 0.003, Cohen’s *d* = − 0.628, 95% CI (− 1.07, − 0.18), adjusted with Bonferroni correction] and States scores [*t*(106) = − 4.24, *p* < 0.001, Cohen’s *d* = − 0.711, 95% CI (− 1.25, − 0.020, adjusted with Bonferroni correction] for the Perceived Unsafety cluster were significantly lower in the Brazilian sample.

#### Non-Refined Factor Scores

**Fig. 3:**
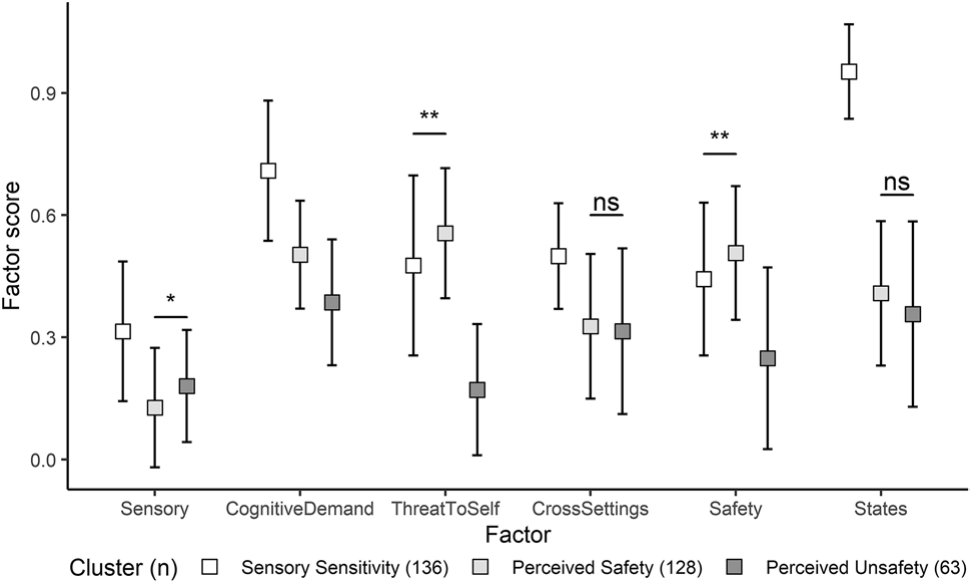
Pairwise comparisons of the three clusters derived from Brazilian non-refined factor scores, luster centroids are displayed as means (squares), with standard deviations as error bars. Unless otherwise specified, all pairwise comparisons within each factor were significant at p < 0.001, adjusted with Tukey’s method, *Ns* not significant; *p < 0.05; **p < 0.01

The responses from the caregivers in Brazil were classified into three clusters using non-refined factor scores. A significant difference in the mean factor scores was found between the clusters [Pillai Trace = 0.729, *F*(6, 320) = 143.71, *p* < 0.001, ω^2^ = 0.724, 95% CI (0.663, 0.767)]. Subsequent univariate (Table 3) and pairwise comparisons (Fig. 3; Supplementary Table 3) revealed patterns of differences between the mean non-refined factor scores of the three clusters that were similar to the patterns identified in the clusters derived from the refined factor scores. The Sensory Sensitivity cluster consisted of relatively high mean scores across factors and the Perceived Safety cluster demonstrated higher mean scores specifically in the Threat to Self and Safety factors. Notably however, the mean Cross-settings factor score of the Perceived Unsafety cluster was relatively low.

**Table 3:** Cluster centroids of the three-cluster solution derived from Brazilian non-refined factor scores

Factor	Cluster Mean (*SD*)			Welch’s *F*	ω^2^	95% CI	Post-hoc summary ^a^
	SS (*n* = 136)	PS (*n* = 128)	PU (*n* = 63)				
Sensory	0.31 (0.17)	0.13 (0.15)	0.18 (0.14)	*F*(2, 175) = 46.6 ^***^	0.218	[0.083, 0.364]	1 > 3 > 2
Cognitive demand	0.71 (0.17)	0.50 (0.13)	0.39 (0.15)	*F*(2, 164) = 101.3 ^***^	0.380	[0.218, 0.526]	1 > 2 > 3
Threat to Self	0.48 (0.22)	0.56 (0.16)	0.17 (0.16)	*F*(2, 174) = 122.4 ^***^	0.426	[0.268, 0.563]	2 > 1 > 3
Cross-settings	0.50 (0.13)	0.33 (0.18)	0.32 (0.20)	*F*(2, 149) = 50.8 ^***^	0.233	[0.085, 0.392]	1 > 2, 3
Safety	0.44 (0.19)	0.51 (0.16)	0.25 (0.22)	*F*(2, 156) = 33.3 ^***^	0.165	[0.042, 0.315]	2 > 1 > 3
States	0.95 (0.12)	0.41 (0.18)	0.36 (0.23)	*F*(2, 142) = 535.5 ^***^	0.766	[0.655, 0.841]	1 > 2, 3

*SS* sensory sensitivity; *PS* perceived safety; *PU* perceived unsafety, p and confidence intervals adjusted with Bonferroni correction, ***p < 0.001

^a^Pairwise Games-Howell tests adjusted with Tukey’s method

The cluster structure derived from the Brazilian non-refined factor scores was compared to classification of the Brazilian responses based on centroids derived from the non-refined factor scores of the English sample to evaluate the utility and generalisability of the cluster centroids. Classification agreement between the clustering methods was poor, as cluster agreement was achieved for 146 of the Brazilian responses (44.6%; Cohen’s unweighted κ = 0.24, 95% CI [0.18, 0.30]).

## Discussion

The present study sought to identify cross-cultural differences in the patterns of contexts associated with emotional outbursts experienced by children and young people with neurodevelopmental disorders in Brazil versus the patterns derived from children and young people using the English version of the Emotional Outburst Questionnaire. In order to facilitate this goal, a Brazilian Portuguese version of the Emotional Outburst Questionnaire was developed. The contextual items of the Brazilian Portuguese version of the questionnaire could be organised into a latent six-factor solution comparable to that identified in the English version. This new factor structure, which involved two additional loadings, appeared to be measurement invariant across the two versions of the questionnaire, such that the responses from the two samples could be represented by the same factor structure and an equal set of factor loadings. Based on these contextual factors, the Brazilian responses were divided into three clusters with distinct patterns of contexts, which resembled the patterns that had previously been identified using the English version of the questionnaire.

### Cultural Differences in the Emotional Outburst Questionnaire

The confirmatory analyses from this study contributed to the validation of a variant of the factor structure underlying the contextual items of the Emotional Outburst Questionnaire, which included the additional loadings from items concerning familiar people and settings onto the Safety factor, which originally consisted of items that would more directly contribute to the perceived safety of an environment (e.g., a private setting or a person that the individual liked; Supplementary Fig. 2 & 3). Although these modifications were primarily data-driven, they nevertheless appeared to be theoretically consistent, as individuals may be more likely to perceive familiar contexts as safe (Brosschot et al., 2018). The measurement invariance of the contextual items in the questionnaire provided further evidence regarding the robustness of the measure across samples and across cultures. Within the field, the ability to compare results across studies of emotional outbursts in people with neurodevelopmental disorders has been limited by the range of approaches that have been used to systematically characterise outbursts in previous studies (e.g., Beauchamp-Châtel et al., 2019; Rice et al., 2018; Tunnicliffe et al., 2014), which has further precluded the consideration of the impact of culture on the aetiology of emotional outbursts. The Emotional Outburst Questionnaire may be able to overcome this barrier and enable direct comparisons across studies utilising the questionnaire and across cultures, but further validation of both the contextual items and the remaining items is warranted.

### The Development of a Culturally Sensitive Framework of Emotional Outbursts

The adaptation of the questionnaire into Brazilian Portuguese may facilitate future efforts to investigate emotional outbursts experienced by people in Brazil, a population for whom information regarding outbursts is scarce. Indeed, such endeavours will be critical in refining the aetiological account of emotional outbursts into a culturally sensitive framework, as the majority of the previous literature regarding outbursts has been based on a Western perspective. Cross-cultural comparisons between individuals from additional countries may further reveal the relevance of cultural factors, such as the orientation towards social independence versus interdependence, in the manifestation of emotional outbursts. Furthermore, the inclusion of culturally sensitive measures in these comparisons may simultaneously identify sources of between-group differences and capture within-group variability, which may both be relevant to the observed heterogeneity of emotional outbursts. For example, in regard to cross-cultural comparisons of emotional outbursts between children and young people in Brazil and the UK, it may be beneficial to include the Beliefs about Emotions Scale (Rimes & Chalder, 2010), as the measure appeared to capture differences between how adults in the UK and Brazil conceptualise the beliefs of the acceptability of negative emotion expression (Mograbi et al., 2018).

The three clusters derived from the refined factor scores of the Brazilian responses appeared to largely resemble the clusters previously identified in the English study, in which the clusters were distinguished by (1) high mean scores across contextual factors; (2) high mean Safety score; and (3) high mean Cross-settings score. The similarities across the samples indicate that these clusters may represent – regardless of the country – cross-cultural pathways that describe the variable aetiology of emotional outbursts, which could form the basis of pathway-specific interventions. Additional cross-cultural validation of the Emotional Outburst Questionnaire may facilitate the use of the questionnaire as a screening tool to support the development and delivery of these interventions by ensuring that the outbursts of individuals are classified in a culturally sensitive manner. Furthermore, future work should assess the replicability of the cluster structure of responses in samples from additional cultures so that the potential generalisability of the present framework could be appraised. If the overall pattern of pathways were indeed found to be cross-culturally consistent, fine-grained cultural differences could nevertheless add a degree of variability to the overall pattern of contexts associated with each pathway (e.g., Caron et al., 2012; Gilbert, 2014; Hull et al., 2020).

Indeed, several differences between the clusters derived from refined factor scores from the two samples were identified in this study. One notable difference was the relatively high mean Threat to Self score for the Perceived Safety cluster in the responses from caregivers in Brazil. This difference may be related to the motivation for a person to mask their emotions to hide characteristics that may be perceived by others as less socially desirable (e.g., Cook et al., 2021). Therefore, a person who is motivated to maintain a socially desirable impression on others by masking their emotions may also be more likely to react negatively in the form of an emotional outburst if their self-image or self-esteem is threatened. The prominence of the mean Threat to Self score in the Perceived Safety cluster of the Brazil sample appears to be consistent with a cultural perspective, as individuals from Brazil may place more value on how they are perceived by others within their community, and the individuals may also receive more prejudice and stigma from the community that may negatively impact their self-esteem (Dessen & Torres, 2019; Paula et al., 2020).

A second difference was that the mean Cross-settings score for the Perceived Unsafety cluster was not as high in the Brazilian responses compared to that of the corresponding cluster in the responses to the English version of the questionnaire. Given that there is less social integration into the wider community for individuals with ASD in Brazil, contexts associated with low perceived safety may be more widespread, such that the Cross-settings factor of the questionnaire could not adequately capture these contexts in the Brazilian responses (Gomes et al., 2015; Weissheimer et al., 2021). Additionally, it is possible that this difference in mean Cross-settings score stemmed from the subjective nature of safety perception. In support of the importance of the subjectivity of contexts in the manifestation of emotional outbursts, a previous study involving adolescents with ASD from the UK reported that contrary to expectations, one participant regarded unpredictable changes to routines as positive rather than negative (Acker et al., 2018). The authors identified that instead of the unpredictability of situations, the perception of being pressurised or rushed was more salient to the manifestation of emotional outbursts for the young people in the study (Acker et al., 2018). Therefore, the investigation of safety perception in relation to emotional outbursts may benefit from further qualitative work with young people who experience outbursts to explore the salience and cross-cultural differences of this relationship.

In contrast to the English study, which had found associations of autism spectrum disorder with the Sensory Sensitivity cluster and intellectual disability with the Perceived Unsafety cluster (Chung et al., 2022), no such associations were identified in the responses from caregivers in Brazil. This lack of association between cluster membership and diagnostic status may be due to differences in the composition of diagnoses in the two samples, as the proportion of young people with a diagnosis of autism spectrum disorder was greater in the Brazilian sample than the English sample, whereas the proportion of young people with intellectual disability was lower in the Brazilian sample (Supplementary Table 1). Regarding other demographic factors in the present study, young people in the Perceived Safety cluster were observed to be less likely to receive medication for outbursts and more likely to have accessed other forms of social or psychological support. These discrepancies in access to medication and other forms of support may potentially stem from individuals in the Perceived Safety cluster having a higher level of adaptive functioning compared to individuals in other clusters, as the underlying mechanism for the Perceived Safety cluster is hypothesised to involve the suppression of negative emotions outside of safe settings, which may require greater regulatory ability to achieve (Chung et al., 2022). Therefore, young people in the Perceived Safety cluster may be more likely to receive psychological as opposed to pharmacological interventions, as some psychological interventions for challenging behaviours have been found to be more effective for people with greater adaptive functioning (e.g., mindfulness training; for recent review, see Woodcock & Blackwell, 2020). From a cultural perspective, it is also possible that families are less likely to access medication over other forms of support for emotional outbursts because the impact of the outbursts experienced by young people in the Perceived Safety cluster may be perceived to be lower in the context of a relatively interdependent culture, as these outbursts are mostly experienced in safe settings such as at home or in a private environment, rather than in contexts where unfamiliar community members may be present.

In terms of classification using the non-refined factor scores from the Brazilian responses, the distinguishing features of the centroids for the Sensory Sensitivity and Perceived Safety clusters were maintained, but the mean Cross-settings score for the Perceived Unsafety cluster was not prominent. The use of unweighted non-refined factor scores would be preferable to regression-based refined factor scores, as it would allow new responses to be scored and classified without the prerequisite of conducting factor and cluster analyses, which would be beneficial for studies with smaller samples or those focusing on specific subsets of emotional outbursts. However, the present study demonstrated that this procedure may not be feasible, as the non-refined factor scores calculated for responses from Brazilian caregivers could not be reliably classified into existing clusters from the English study. It is possible that the loss of information from the use of unweighted factor scores hinders reliable classification in this way, so a compromise could potentially be achieved through the use of factor scores weighted by factor loadings (DiStefano et al., 2009). Additionally, there may be inherent cross-cultural variability in the centroids of the clusters that prevent cross-cultural classification. Therefore, further within- and cross-cultural comparisons should be conducted to investigate whether standardised cluster centroids could be derived either within or across cultures.

### Limitations and Future Directions

The present study was limited by the diagnostic homogeneity of the young people within the sample, which precluded further investigations into potential diagnostic differences in cluster membership. However, the converging evidence between the English study, which included higher heterogeneity in terms of diagnoses of young people, and the current study suggest that the proposed pathways may indeed be transdiagnostic (Chung et al., 2022). Future studies could address this question more directly by using a between-groups design to compare the patterns of outbursts between individuals with different diagnoses. The broader demographic differences between the two samples should be considered and potentially controlled for in subsequent studies, as the potential effects of these demographic differences could not be accounted for in the present study. For example, investigations focusing on the emotional outbursts experienced by young people in the upper age range of the present study may be beneficial, as they were less represented in the present sample. Furthermore, as the Emotional Outburst Questionnaire is an informant-report measure, the potential effect of caregiver educational level on the accuracy of responses should be examined (e.g., Van Roy et al., 2010). A critical avenue for future work lies in assessing the associations between cluster membership and measures of differences in the proposed underlying mechanisms to further delineate the aetiology of emotional outbursts.

## Conclusions

This study demonstrated that the contextual items of the Brazilian Portuguese version of the Emotional Outburst Questionnaire could be organised into a latent six-factor structure, which was measurement invariant across the Brazilian Portuguese and English versions of the questionnaire. Three clusters were generated from the responses from caregivers in Brazil, which were found to be comparable to corresponding clusters from a culturally distinct sample in terms of the distinguishing features of each cluster. The present results suggest that these clusters may represent aetiological pathways of emotional outbursts that are both transdiagnostic and cross-cultural.

### Supplementary Information

Below is the link to the electronic supplementary material.

Supplementary file1 (DOCX 183 kb)

## Data Availability

Data and code for this study is openly available at https://osf.io/6pvea/.

## Declarations

### Concent to Participate

Participants provided informed consent prior to their participation.

## References

[CR1] Acker L, Knight M, Knott F (2018). ‘Are they just gonna reject me?’ male adolescents with autism making sense of anxiety: An interpretative phenomenological analysis. Research in Autism Spectrum Disorders.

[CR2] Aldao A, Sheppes G, Gross JJ (2015). Emotion regulation FLEXIBILITY. Cognitive Therapy and Research.

[CR3] Astle DE, Holmes J, Kievit R, Gathercole SE (2022). Annual research review: The transdiagnostic revolution in neurodevelopmental disorders. Journal of Child Psychology and Psychiatry and Allied Disciplines.

[CR4] Balbueno, B. (2021). *Processo de adaptação da versão brasileira do emotional outburst questionnaire: estudo piloto com pessoas com Síndrome de Down e Transtorno do Espectro Autista*. https://dspace.mackenzie.br/handle/10899/28630

[CR5] Beauchamp-Châtel A, Courchesne V, Forgeot d’Arc B, Mottron L (2019). Are tantrums in autism distinct from those of other childhood conditions? a comparative prevalence and naturalistic study. Research in Autism Spectrum Disorders.

[CR6] Bronfenbrenner U, Morris PA, Damon W, Lerner R (2006). The Bioecological Model of Human Development. Handbook of child psychology: Theoretical models of human development.

[CR7] Brosschot JF, Verkuil B, Thayer JF (2018). Generalized unsafety theory of stress: Unsafe environments and conditions, and the default stress response. International Journal of Environmental Research and Public Health.

[CR8] Caron KG, Schaaf RC, Benevides TW, Gal E (2012). Cross-cultural comparison of sensory behaviors in children with autism. American Journal of Occupational Therapy.

[CR9] Chen FF (2007). Sensitivity of goodness of fit indexes to lack of measurement invariance. Structural Equation Modeling.

[CR10] Chung JCY, Mevorach C, Woodcock KA (2022). Establishing the transdiagnostic contextual pathways of emotional outbursts. Scientific Reports.

[CR11] Colombo D, Fernández-Álvarez J, Suso-Ribera C, Cipresso P, Valev H, Leufkens T, Sas C, Garcia-Palacios A, Riva G, Botella C (2020). The need for change: Understanding emotion regulation antecedents and consequences using ecological momentary assessment. Emotion.

[CR12] Cook J, Crane L, Bourne L, Hull L, Mandy W (2021). Camouflaging in an everyday social context: An interpersonal recall study. Autism.

[CR13] Corapci F, Friedlmeier W, Benga O, Strauss C, Pitica I, Susa G (2018). Cultural socialization of toddlers in emotionally charged situations. Social Development.

[CR14] Cressey H, Oliver C, Crawford H, Waite J (2019). Temper outbursts in lowe syndrome: Characteristics, sequence, environmental context and comparison to Prader-Willi syndrome. Journal of Applied Research in Intellectual Disabilities.

[CR15] Dammeyer J, Chapman M (2018). A national survey on violence and discrimination among people with disabilities. BMC Public Health.

[CR16] Dessen MA, Torres CV (2019). Family and socialization factors in Brazil: An overview. Online Readings in Psychology and Culture.

[CR17] DiStefano, C., Zhu, M., & Mîndrilǎ, D. (2009). Understanding and using factor scores: Considerations for the applied researcher. *Practical Assessment, Research and Evaluation*, *14*(20), 20. https://scholarworks.umass.edu/pare/vol14/iss1/20

[CR18] Einfeld SL, Tonge BJ (1995). The developmental behavior checklist: The development and validation of an instrument to assess behavioral and emotional disturbance in children and adolescents with mental retardation. Journal of Autism and Developmental Disorders.

[CR19] Ford BQ, Mauss IB (2015). Culture and emotion regulation. Current Opinion in Psychology.

[CR20] Friedlmeier W, Çorapçi F, Benga O, Jensen LA (2014). Early Emotional Development in Cultural Perspective. The Oxford Handbook of Human Development and Culture.

[CR21] Gilbert P (2014). The origins and nature of compassion focused therapy. British Journal of Clinical Psychology.

[CR22] Gomes PTM, Lima LHL, Bueno MKG, Araújo LA, Souza NM (2015). Autism in Brazil: A systematic review of family challenges and coping strategies. Jornal De Pediatria (versão Em Português).

[CR23] Harris PA, Taylor R, Thielke R, Payne J, Gonzalez N, Conde JG (2009). Research electronic data capture (REDCap)-A metadata-driven methodology and workflow process for providing translational research informatics support. Journal of Biomedical Informatics.

[CR24] Harris PA, Taylor R, Minor BL, Elliott V, Fernandez M, O’Neal L, McLeod L, Delacqua G, Delacqua F, Kirby J, Duda SN (2019). The REDCap consortium: Building an international community of software platform partners. Journal of Biomedical Informatics..

[CR25] Hu LT, Bentler PM (1999). Cutoff criteria for fit indexes in covariance structure analysis: Conventional criteria versus new alternatives. Structural Equation Modeling.

[CR26] Hull L, Petrides KV, Mandy W (2020). The Female autism phenotype and camouflaging: A narrative review. In Review Journal of Autism and Developmental Disorders..

[CR27] International Test Commission. (2017). *The ITC Guidelines for Translating and Adapting Tests (Second edition)*. www.InTestCom.org

[CR28] Jorgensen, T. D., Pornprasertmanit, S., Schoemann, A. M., & Rosseel, Y. (2021). *semTools: Useful tools for structural equation modeling*. https://cran.r-project.org/package=semTools

[CR29] Kinnear SH, Link BG, Ballan MS, Fischbach RL (2016). Understanding the experience of stigma for parents of children with autism spectrum disorder and the role stigma plays in families’ lives. Journal of Autism and Developmental Disorders.

[CR30] Kline RB (2016). Principles and Practice of Structural Equation Modeling (Fourth).

[CR31] Lowe K, Felce D (1995). How do careers assess the severity of challenging behaviour? A total population study. Journal of Intellectual Disability Research.

[CR32] Lowe K, Allen D, Jones E, Brophy S, Moore K, James W (2007). Challenging behaviours: Prevalence and topographies. Journal of Intellectual Disability Research.

[CR33] Markus, H. R., & Kitayama, S. (1991). Culture and the Self: Implications for Cognition, Emotion, and Motivation. *Psychological Review*, *98*(2), 224–253. http://psycnet.apa.orgjournals/rev/98/2/224

[CR34] Markus HR, Kitayama S (2010). Cultures and selves: A cycle of mutual constitution. Perspectives on Psychological Science.

[CR35] Matsumoto D, Yoo SH, Nakagawa S, Alexandre J, Altarriba J, Anguas-Wong AM, Arriola M, Bauer LM, Bond MH, Cabecinhas R, Chae J, Comunian AL, DeGere DN, de Melo Garcia Bley, L., Fok, H. K., Friedlmeier, W., Garcia, F. M., Ghosh, A., Granskaya, J. V., … Yoo, S. H. (2008). Culture, emotion regulation, and adjustment. Journal of Personality and Social Psychology.

[CR36] Mazefsky CA, Yu L, White SW, Siegel M, Pilkonis PA (2018). The emotion dysregulation inventory: Psychometric properties and item response theory calibration in an autism spectrum disorder sample. Autism Research.

[CR37] Mograbi DC, Indelli P, Lage CA, Tebyriça V, Landeira-Fernandez J, Rimes KA (2018). Cross-cultural adaptation and validation of the Brazilian version of the beliefs about emotions scale. Trends in Psychiatry and Psychotherapy.

[CR38] Montaque I, Dallos R, McKenzie B (2018). “It feels like something difficult is coming back to haunt me”: An exploration of ‘meltdowns’ associated with autistic spectrum disorder from a parental perspective. Clinical Child Psychology and Psychiatry.

[CR39] Myrbakk E, Tetzchner SV (2008). The prevalence of behavior problems among people with intellectual disability living in community settings?. Journal of Mental Health Research in Intellectual Disabilities.

[CR40] Paula CS, Cukier S, Cunha GR, Irarrázaval M, Montiel-Nava C, Garcia R, Rosoli A, Valdez D, Bordini D, Shih A, Garrido G, Rattazzi A (2020). Challenges, priorities, barriers to care, and stigma in families of people with autism: Similarities and differences among six Latin American countries. Autism.

[CR41] R Core Team. (2021). *R: A Language and Environment for Statistical Computing*. https://www.r-project.org/

[CR42] Ramzan N, Amjad N (2017). Cross cultural variation in emotion: A systematic review. Annals of KEMU.

[CR43] Rice LJ, Woodcock KA, Einfeld SL (2018). The characteristics of temper outbursts in Prader-Willi syndrome. American Journal of Medical Genetics, Part A.

[CR44] Rimes KA, Chalder T (2010). The Beliefs about Emotions Scale: Validity, reliability and sensitivity to change. Journal of Psychosomatic Research.

[CR45] Rosseel Y (2012). lavaan: An R package for structural equation modeling. Journal of Statistical Software..

[CR46] Ryan S (2010). “Meltdowns”, surveillance and managing emotions; going out with children with autism. Health and Place.

[CR47] Schermelleh-Engel K, Moosbrugger H, Müller H (2003). Evaluating the fit of structural equation models: Tests of significance and descriptive goodness-of-fit measures. Methods of Psychological Research Online.

[CR48] Svetina D, Rutkowski L, Rutkowski D (2020). Multiple-group invariance with categorical outcomes using updated guidelines: An illustration using mplus and the lavaan/semtools packages. Structural Equation Modeling.

[CR49] Tunnicliffe P, Oliver C (2011). Phenotype-environment interactions in genetic syndromes associated with severe or profound intellectual disability. Research in Developmental Disabilities.

[CR50] Tunnicliffe P, Woodcock K, Bull L, Oliver C, Penhallow J (2014). Temper outbursts in Prader-Willi syndrome: Causes, behavioural and emotional sequence and responses by carers. Journal of Intellectual Disability Research.

[CR51] van de Schoot R, Lugtig P, Hox J (2012). A checklist for testing measurement invariance. European Journal of Developmental Psychology.

[CR52] Van Doren N, Zainal NH, Newman MG (2021). Cross-cultural and gender invariance of emotion regulation in the United States and India. Journal of Affective Disorders.

[CR53] Van Roy B, Groholt B, Heyerdahl S, Clench-Aas J (2010). Understanding discrepancies in parent-child reporting of emotional and behavioural problems: Effects of relational and socio-demographic factors. BMC Psychiatry.

[CR54] Wakschlag LS, Choi SW, Carter AS, Hullsiek H, Burns J, McCarthy K, Leibenluft E, Briggs-Gowan MJ (2012). Defining the developmental parameters of temper loss in early childhood: Implications for developmental psychopathology. Journal of Child Psychology and Psychiatry and Allied Disciplines.

[CR55] Wakschlag LS, Briggs-Gowan MJ, Choi SW, Nichols SR, Kestler J, Burns JL, Carter AS, Henry D (2014). Advancing a multidimensional, developmental spectrum approach to preschool disruptive behavior. Journal of the American Academy of Child and Adolescent Psychiatry.

[CR56] Ward JH (1963). Hierarchical Grouping to Optimize an Objective Function. Journal of the American Statistical Association.

[CR57] Weissheimer G, de Mazza V, A., Santana, J. M., Ruthes, V. B. T. N. M., & Freitas, C. A. S. L. (2021). Information demands from families of children with Autism Spectrum Disorder. Revista Brasileira De Enfermagem.

[CR58] White SW, Mazefsky CA, Dichter GS, Chiu PH, Richey JA, Ollendick TH (2014). Social-cognitive, physiological, and neural mechanisms underlying emotion regulation impairments: Understanding anxiety in autism spectrum disorder. International Journal of Developmental Neuroscience.

[CR59] Woodcock KA, Blackwell S (2020). Psychological treatment strategies for challenging behaviours in neurodevelopmental disorders: What lies beyond a purely behavioural approach?. Current Opinion in Psychiatry.

[CR60] Woodcock KA, Oliver C, Humphreys GW (2011). The relationship between specific cognitive impairment and behaviour in Prader-Willi syndrome. Journal of Intellectual Disability Research.

[CR61] Wu H, Estabrook R (2016). Identification of confirmatory factor analysis models of different levels of invariance for ordered categorical outcomes. Psychometrika.

