# Supplementary material for: Cross-Cultural Comparison of the Contexts Associated with Emotional Outbursts
Source: J Autism Dev Disord. 2022 Aug 19;53(11):4229–42. doi: 10.1007/s10803-022-05708-7 (PMC10539459; doi:10.1007/s10803-022-05708-7)
Supplement: Supplementary file 1 — Supplementary file1 (DOCX 183 kb) [file 10803_2022_5708_MOESM1_ESM.docx]

**Supplementary Information**

**Supplementary Table 1** Comparison of demographic information between caregivers of the English and Brazilian samples.

| Variable | Sample | | Statistic | Effect size ^a^ | 95% CI |
| --- | --- | --- | --- | --- | --- |
|  | English | Brazilian |  |  |  |
| *N* | 268 | 327 |  |  |  |
| Age |  |  |  |  |  |
| Mean | 13.5 | 10.7 | *t*(468) = 7.5 ^***^ | 0.626 | [0.46, 0.79] |
| *SD* | 5.2 | 3.7 |  |  |  |
| Gender (*n* [%]) ^b^ |  |  | χ^2^ (1) = 16.5 ^***^ | 0.170 | [0.096, 0.251] |
| Male | 162 (60.4) | 250 (76.5) |  |  |  |
| Female | 105 (39.2) | 77 (23.5) |  |  |  |
| Other | 1 (0.4) | 0 (0) |  |  |  |
| Diagnosis (*n* [%]) ^c^ |  |  |  |  |  |
| Autism spectrum disorder | 132 (49.3) | 272 (83.2) | χ^2^ (1) = 76.2 ^***^ | 0.362 | [0.284, 0.431] |
| Down’s syndrome | 4 (1.5) | 51 (15.6) | χ^2^ (1) = 33.3 ^***^ | 0.242 | [0.189, 0.294] |
| Intellectual disability | 77 (28.7) | 29 (8.9) | χ^2^ (1) = 38.3 ^***^ | 0.258 | [0.181, 0.333] |
| Medication (*n* [%]) ^d^ |  |  | χ^2^ (1) = 2.7 | 0.071 | [0.006, 0.145] |
| Yes | 70 (26.2) | 107 (32.7) |  |  |  |
| Access to support (*n* [%]) ^e^ |  |  | χ^2^ (1) = 71.5 ^***^ | 0.351 | [0.270, 0.426] |
| Yes | 130 (49.2) | 54 (16.5) |  |  |  |
| Schooling or employment (*n* [%]) ^f^ |  |  | χ^2^ (3) = 51.1 ^***^ | 0.298 | [0.229, 0.375] |
| Mainstream school | 146 (54.9) | 262 (80.1) |  |  |  |
| Special school | 68 (25.6) | 51 (15.6) |  |  |  |
| Further education | 11 (4.1) | 1 (0.3) |  |  |  |
| Higher education | 2 (0.8) | 1 (0.3) |  |  |  |
| Employment preparation | 4 (1.5) | 4 (1.2) |  |  |  |
| Employed | 5 (1.9) | 0 (0) |  |  |  |
| Unemployed | 30 (11.3) | 7 (2.1) |  |  |  |
| Statement of special educational needs or educational plan (*n* [%]) ^g^ |  |  | χ^2^ (1) = 0.1 | 0.016 | [0.001, 0.102] |
| Yes | 160 (59.7) | 194 (59.3) |  |  |  |
| Trauma (*n* [%]) ^h^ |  |  | χ^2^ (1) = 101 ^***^ | 0.474 | [0.379, 0.559] |
| Yes | 73 (48.3) | 25 (7.6) |  |  |  |

^a^ Cohen’s *d* for Welch’s t-test and Cramer’s V for χ^2^ tests.

^b^ Non-binary response from English sample excluded for χ^2^ test.

^c^ Proportion of young people with each diagnosis. Each caregiver could indicate more than one diagnosis for the multiple-choice question in the survey.

^d^ One response missing in English sample.

^e^ Four responses missing in English sample.

^f^ Two responses missing in English sample; one response missing in Brazilian sample. Responses indicating *Higher education*, *Employment preparation*, or *Employed* excluded for χ^2^ test.

^g^ Four responses missing in English sample.

^h^ One hundred and seventeen responses missing and four selected *Prefer not to say* in English sample; 11 responses missing and four selected *Prefer not to say* in Brazilian sample. Responses indicating *Prefer not to say* excluded for χ^2^ test.

^***^ *p* < 0.001.

**Supplementary Table 2** Differences in cluster centroids derived from Brazilian refined factor scores.

| Factor | Mean difference (95% CI) | | |
| --- | --- | --- | --- |
|  | SS-PS | SS-PU | PS-PU |
| Sensory | -1.50 [-1.69, -1.31] ^***^ | -0.90 [-1.09, -0.72] ^***^ | 0.59 [0.39, 0.80] ^***^ |
| Cognitive Demand | -1.06 [-1.23, -0.88] ^***^ | -1.50 [-1.70, -1.30] ^***^ | -0.44 [-0.64, -0.24] ^***^ |
| Threat to Self | 0.30 [0.09, 0.51] ^**^ | -1.05 [-1.28, -0.81] ^***^ | -1.34 [-1.56, -1.13] ^***^ |
| Cross-settings | -1.58 [-1.76, -1.41] ^***^ | -0.80 [-1.01, -0.59] ^***^ | 0.78 [0.56, 1.01] ^***^ |
| Safety | 0.80 [0.57, 1.03] ^***^ | -0.53 [-0.78, -0.29] ^***^ | -1.33 [-1.58, -1.08] ^***^ |
| States | -1.36 [-1.52, -1.21] ^***^ | -1.25 [-1.44, -1.06] ^***^ | 0.11 [-0.08, 0.31] |

SS, Sensory Sensitivity; PS, Perceived Safety; PU, Perceived Unsafety.

*p* and confidence intervals adjusted with Tukey’s method.

** *p* < 0.01; *** *p* < 0.001.

**Supplementary Table 3** Differences in cluster centroids derived from Brazilian non-refined factor scores.

| Factor | Mean difference (95% CI) | | |
| --- | --- | --- | --- |
|  | SS-PS | SS-PU | PS-PU |
| Sensory | -0.19 [-0.23, -0.14] ^***^ | -0.13 [-0.19, -0.08] ^***^ | 0.05 [0.00, 0.10] ^*^ |
| Cognitive Demand | -0.21 [-0.25, -0.16] ^***^ | -0.32 [-0.38, -0.27] ^***^ | -0.12 [-0.17, -0.06] ^***^ |
| Threat to Self | 0.08 [0.02, 0.13] ^**^ | -0.31 [-0.37, -0.24] ^***^ | -0.38 [-0.44, -0.33] ^***^ |
| Cross-settings | -0.17 [-0.22, -0.13] ^***^ | -0.18 [-0.25, -0.12] ^***^ | -0.01 [-0.08, 0.06] |
| Safety | 0.06 [0.01, 0.12] ^**^ | -0.19 [-0.27, -0.12] ^***^ | -0.26 [-0.33, -0.18] ^***^ |
| States | -0.54 [-0.59, -0.50] ^***^ | -0.60 [-0.67, -0.52] ^***^ | -0.05 [-0.13, 0.03] |

SS, Sensory Sensitivity; PS, Perceived Safety; PU, Perceived Unsafety.

*p* and confidence intervals adjusted with Tukey’s method.

* *p* < 0.05; ** *p* < 0.01; *** *p* < 0.001.

**Supplementary Fig. 1** Age frequency distribution of children and young people in the Brazilian sample.

**
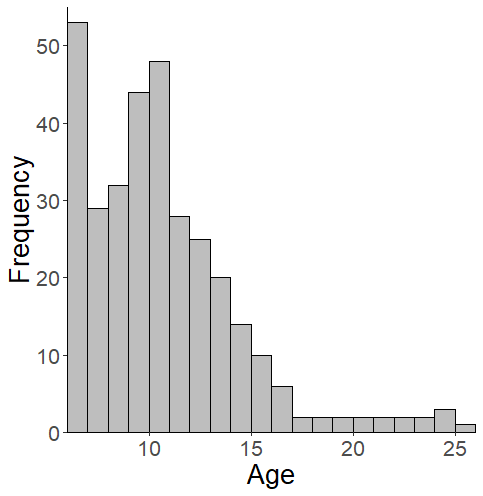
**

**Supplementary Fig. 2** Six-factor structure of contextual items of the Emotional Outburst Questionnaire based on responses from the previous English study.
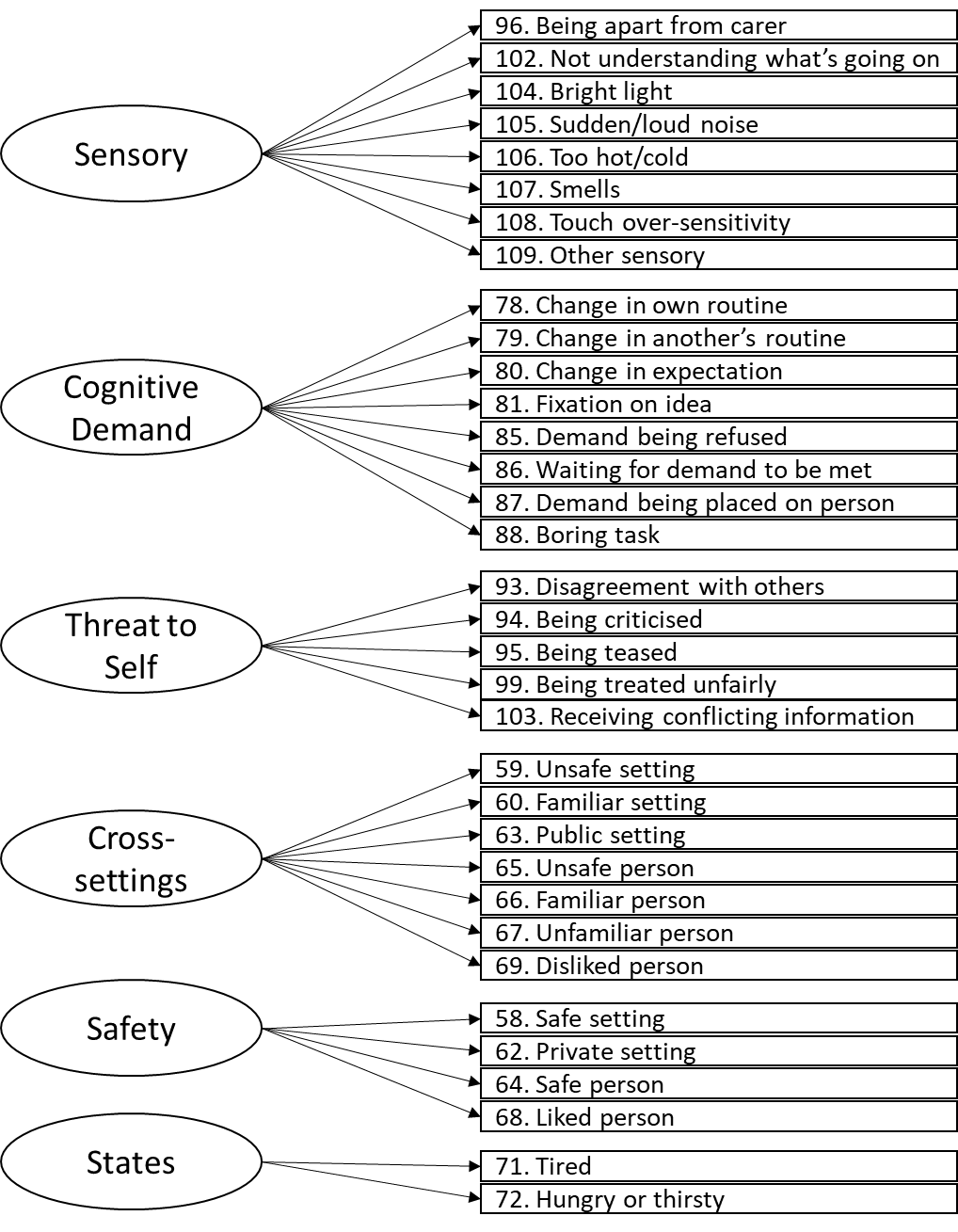


Ellipses represent factors; rectangles represent contextual items.

**Supplementary Fig. 3** Revised six-factor structure of contextual items of the Emotional Outburst Questionnaire based on responses from the present study.
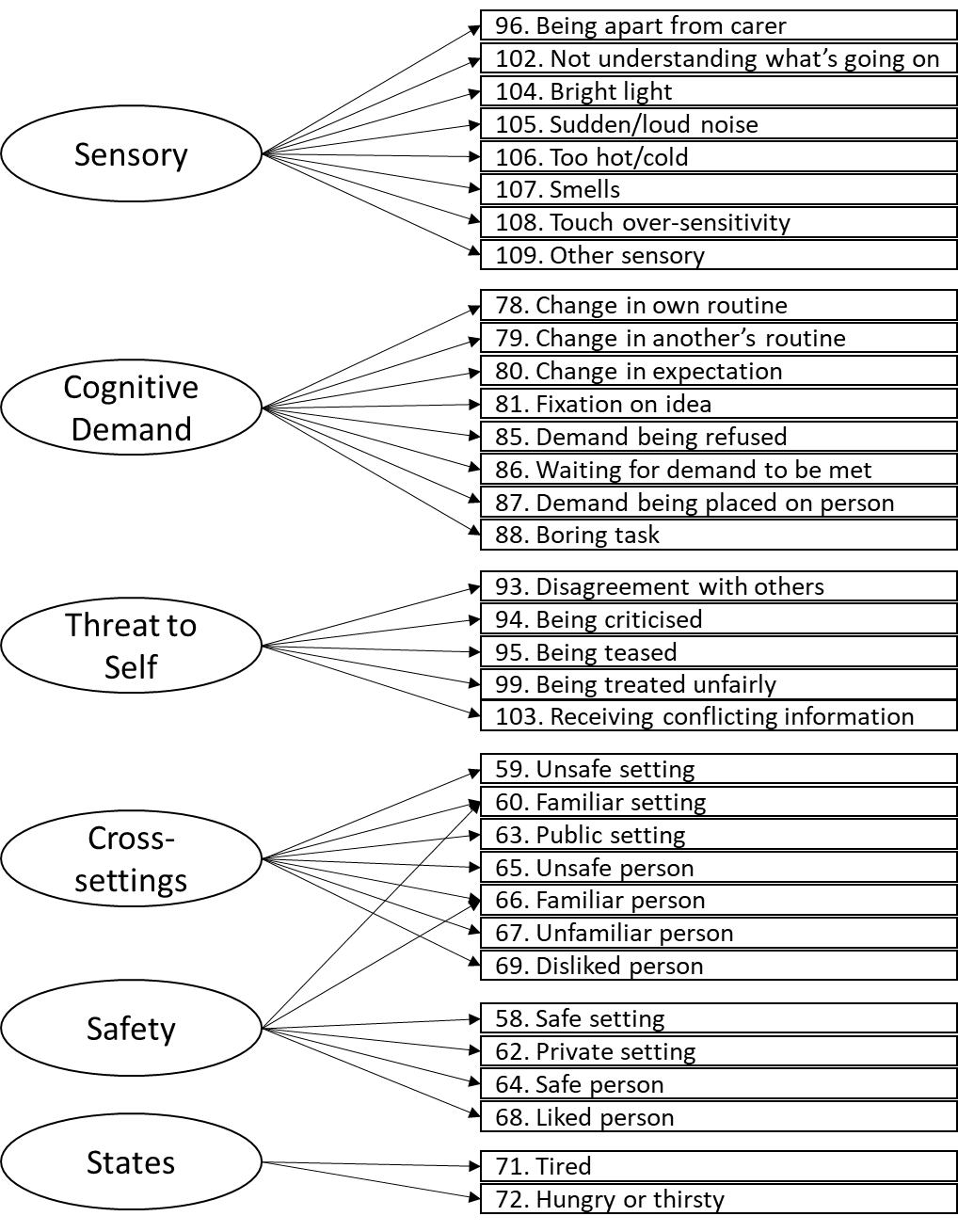
 Ellipses represent factors; rectangles represent contextual items. Covariances between factors and variances of each contextual item are not shown in the figure but were estimated by the models specified in the present study.
